# The positive role of vitronectin in radiation induced lung toxicity: the in vitro and in vivo mechanism study

**DOI:** 10.1186/s12967-018-1474-y

**Published:** 2018-04-16

**Authors:** Tian-Le Shen, Mi-Na Liu, Qin Zhang, Wen Feng, Wen Yu, Xiao-Long Fu, Xu-Wei Cai

**Affiliations:** 1 Department of Radiation Oncology, Shanghai Chest Hospital, Shanghai Jiao Tong University, 241 Huai Hai West Road, Shanghai, 200030 China; 2 Department of Radiation Oncology, Shanghai Cancer Center, Fudan University, Shanghai, 200032 China

**Keywords:** Radiation therapy, Radiation induced lung fibrosis, Vitronectin, Fibroblasts, TGF-β

## Abstract

**Background:**

Radiation-induced lung toxicity (RILT) is a severe complication of radiotherapy in patients with thoracic tumors. Through proteomics, we have previously identified vitronectin (VTN) as a potential biomarker for patients with lung toxicity of grade ≥ 2 radiation. Herein, we explored the molecular mechanism of VTN in the process of RILT.

**Methods:**

In this study, lentivirus encoding for VTN and VTN-specific siRNA were constructed and transfected into the cultured fibroblasts and C57BL mice. Real-time PCR, western blot and ELISA were used to examine expression of collagens and several potential proteins involved in lung fibrosis. Hematoxylin–eosin and immunohistochemical staining were used to assess the fibrosis scores of lung tissue from mice received irradiation.

**Results:**

The expression of VTN was up-regulated by irradiation. The change trend of collagens, TGF-β expression and p-ERK, p-AKT, and p-JNK expression levels were positively related with VTN mRNA level. Furthermore, overexpression of VTN significantly increased the expression level of α-SMA, as well as the degree of lung fibrosis in mice at 8 and 12 weeks post-irradiation. By contrast, siRNA VTN induced opposite results both in vitro and in vivo.

**Conclusions:**

VTN played a positive role in the lung fibrosis of RILT, possibly through modulation of fibrosis regulatory pathways and up-regulating the expression levels of fibrosis-related genes. Taken together, all the results suggested that VTN had a novel therapeutic potential for the treatment of RILT.

**Electronic supplementary material:**

The online version of this article (10.1186/s12967-018-1474-y) contains supplementary material, which is available to authorized users.

## Background

Radiation therapy acts as an effective treatment for thoracic malignant tumor [1, 2]. High radiation dosage could contribute to local tumor control and high survival rate [3]. However, along with the increased radiation dose, the severity of radiation-induced lung injury increased and ultimately resulted in lung fibrosis [4–6]. RILT, as one of the common complications caused by radiotherapy for thoracic tumors, may lead to a progressive and irreversible damage to the pulmonary function of patients, and even to death. Accumulating evidences suggested that RILT is mainly caused by the persistent inflammation after initial injury, which leads to an excessive repair process, characterized by secretion of vast inflammatory factors, cytokines and chemokines, causing accumulation of extracellular components, including hyaluronic acid, fibronectin, proteoglycans, and collagens [7, 8]. However, the specific signaling pathways and genes regulated in this process have not been illuminated clearly. Therefore, it is of great importance to explore the underlying mechanisms of radiation induced lung fibrosis and to develop new treatment strategies for RILT.

Our previous studies screened out some proteins of predictive value on RILT by analyzing the patients’ plasma proteins before and after receiving radiation therapy through proteomic. By comparing non-small cell lung cancer patients, who had developed a grade ≥ 2 radiation-induced lung toxicity (RILT) after receiving radiotherapy, with those who did not receive radiotherapy, higher expression level of vitronectin (VTN) was found in the plasma (p = 0.02) [9]. Therefore, we inferred that the up-regulated basal expression of VTN may be related to RILT.

VTN, also known as S-protein, a kind of glycoprotein found in the extracellular matrix of diverse tissues and circular blood, is implicated in diverse cellular processes including cell spreading, cell adhesion and cell migration [10–12]. It also has been reported that high expression levels of VTN were detected in breath condensates exhaled from patients with interstitial pneumonia [13]. Furthermore, the expression level of VTN was also found to be up-regulated in patients with asthma and cirrhotic liver [14, 15]. Even more importantly, Courey et al. observed that the VTN-binding function of PAI-1 exacerbated lung fibrotic processes [16] and a recent proteomic study also revealed VTN could be as an early molecular signature of liver fibrosis in hepatitis C sufferers [17]. In a word, all these studies suggested that there is an essential linkage between VTN and lung fibrosis. However, the effects and mechanisms of the role played by VTN in lung fibrosis are still unknown.

PI3K, AKT1, ERK1 and JNK were reported to be the downstream signals of TGF-β, which could result in human lung parenchymal fibrosis through increased expression in a variety of lung tissues and cellular sources including macrophage and epithelial cells. The PI3K/AKT pathway is an intracellular signaling pathway playing important role in regulating the cell cycle. Therefore, it is directly related to cellular quiescence, proliferation, cancer, and longevity. It has been reported that PI3K/AKT, as upstream of ER stress, could affect lung fibroblast proliferation, resulting in bleomycin-induced pulmonary fibrosis [18]. Inhibition of Extracellular signal-regulated kinase (ERK) was also reported as the improvement of histopathologic changes for lung fibrosis in the lung tissues extracted from bleomycin-induced mice [19]. Besides, the c-Jun N-terminal kinase (JNK) pathway is activated by multiple cytokines and exposure to environmental stress. Its inhibition was also reported to be capable of reducing lung remodeling and pulmonary fibrotic systemic markers [20]. Taken together, we hypothesized that PI3K/AKT, ERK and JNK may all be related with lung fibrosis of RILT.

Bearing all these in mind, in this study, we irradiated lung fibroblasts with different doses to observe the expression changes of VTN and collagens, and to choose optimal experimental condition for constructing RILT cell models. VTN overexpression or knockdown lentivirus vectors were constructed for further researches both in vitro and in vivo. The experimental results suggested that overexpression of VTN promoted the expression of collagen I, collagen III, hydroxyproline, α-SMA and TGF-β in vivo, which further activated the regulatory pathways of lung fibrosis, and eventually aggravated RILT. In a word, this study verified that VTN may be a novel therapeutic target for patients with RILT.

## Methods

### Cell culture

The human fibroblasts WI-38 and IMR-90 cell lines were purchased from American type culture collection (ATCC), and cultured at 37 °C in a humidified atmosphere containing 5% carbon dioxide with F-12K (Hyclone, Beijing, China) supplemented with 20% FBS (Gibco, Invitrogen, Carlsbad, CA, USA).

### Irradiation

The human fibroblasts WI-38 and IMR-90 cells were irradiated with ^137^Cs at the doses of 0 Gy (control), 4, 6, 8, 10 and 12 Gy respectively. Dose rate was 77 cGy/min and SSD was 10 cm.

### Western blotting

According to standard procedures of western blotting, the protein samples extracted from the fibroblasts WI-38 and IMR-90 cells after receiving irradiation which were separated by 10% SDS-PAGE and then transferred to PVDF membranes (Millipore, Billerica, MA). After blocking with 5% non-fat milk in TBST for 1 h at room temperature, these membranes were incubated with the following primary antibodies (1:1000) at 4 °C overnight: anti-VTN monoclonal antibody (mAb) (Abcam, Cambridge, MA, US), anti-collagen I mAb (Abcam, Cambridge, MA, US), anti-collagen III mAb (Abcam, Cambridge, MA, US), anti-α-SMA mAb (CST, Beverly, MA, USA), anti-TGF-β mAb (Biolegend, San Diego, CA, USA), anti-p-ERK mAb (Santa, Santa Cruz, CA, USA), anti-ERK mAb (Pierce, Cruz, CA, USA), anti-p-AKT mAb (CST, Beverly, MA, USA), anti-AKT mAb (CST, Beverly, MA, USA), anti-p-JNK mAb (CST, Beverly, MA, USA), anti-JNK mAb (CST, Beverly, MA, USA), anti-GAPDH mAb (Sigma-Aldrich, St. Louis, MO, USA) and anti-β-actin mAb (Bioworld Technology, Inc. St. Louis, MO, USA). After washing three times with TBST, the membranes were incubated with secondary antibodies conjugated with HRP (1:10,000) for 1 h at room temperature. At last, these membranes were visualized using enhanced chemiluminescence reagents (Pierce, Cruz, CA, USA) according to the manufacturer’s instructions.

### ELISA

The protein levels of VTN, collagen I, collagen III, Hydroxyproline and α-SMA in WI-38 and IMR-90 cells after receiving irradiation were detected with TMB enzyme-linked immunosorbent assay (ELISA) kit (Invitrogen, GIBCO, Carlsbad, CA, USA) according to the manufacturer’s instructions. The absorbance was measured by a microplate reader (Bio-Rad, Hercules, CA, USA).

### RNA extraction and quantitative real-time PCR

Total RNA from cultured WI-38 and IMR-90 cells or tissue samples after received irradiation was extracted by using RNA-Trizol reagent (Invitrogen, Gibco, Carlsbad, CA, USA), and reversely transcribed into cDNA using extraction kit (Amresco, Solon, HO, USA). Primers for quantitative RT-PCR (qRT-PCR) were listed in Additional file 1: Table S1. PCR process and data collection were performed on the 7900HT Fast Real-Time PCR system (Applied Biosystem, Carlsbad, CA, USA) according to the manufacturer’s protocol and GAPDH was used as the reference gene.

### Vector construction, lentivirus production and transduction

The cDNA sequence of VTN was amplified from pGEM-VTN and constructed into the vector pCDH to generate pCDH-VTN. The vector pCDH-VTN went through initial bacterial colony, PCR filtering, doubled digestion and gene sequencing assessment, and then was used to prepare lentivirus by co-transfecting into 293T cells with liposome. Three siRNA sequences targeted at VTN were designed by RNAi designer, and synthesized as follows in Additional file 1: Table S2. The siRNA sequences were inserted into PLVX vector to generate PLVX-si-RNA-VTN. A mixture of pGEM-VTN or PLVX-si-RNA-VTN, psPAX2 and pMDG2 were co-transfected into 293T cells using lipofectamine 2000 reagent to produce lentivirus. WI-38 cells were infected with the recombinant lentivirus-transducing units and 8 μg/mL polybrene (Sigma, St. Louis, MO, USA).

### In vivo study

This study was approved by the Ethic Committee of Shanghai Chest Hospital, Shanghai Jiao Tong University (050432-4-1008A) and all the experiment procedures involved in animals were conducted according to the Guideline of Animal Care and Use Committee of Shanghai Chest Hospital, Shanghai Jiao Tong University. Sixty male C57 black 6 (C57 BL/6) mice with 3 months old which were purchased from the Shanghai Model Organisms Center, Inc., (Shanghai, China) were randomly divided into 5 groups, including normal group (for the mice without irradiation), model group (for the mice with irradiation only), sc-RNA group (for the mice with empty virus with irradiation), OE-VTN group (for the mice infected with VTN-overexpressing lentivirus with irradiation) and the siRNA group (for the mice infected with VTN-si-RNA lentivirus with irradiation). Saline (for model group), empty lentivirus (the vacant group as negative control). VTN-overexpressing lentivirus (for the OE-VTN group) and VTN-si-RNA lentivirus (for the siRNA group) were given to C57 BL/6 mice (12-week-old C57 BL/6, male, 12 mice/group, 1 × 10^7^ TU/mL) via tail vein injection, respectively. After transfected for 48 h, the mice in the 4 irradiation groups received a single dose of 12 Gy irradiation at the whole semi-thorax by using 6 MeV linear accelerator (ClinaciX, Varian), once only a mouse could receive irradiation. SSD was 100 cm, dose rate was 600 cGy/min, and irradiation length was 1 cm. After 8 or 12 weeks of irradiation, mice were sacrificed. Their lungs were obtained surgically and then subjected to hematoxylin and eosin (H&E) staining. The levels of hydroxyproline were evaluated by alkaline hydrolysed sample assay according to protocol.

### Immunohistochemical staining and fibrosis score

Immunohistochemical staining was operated according to standard procedures. In brief, the lung tissues from all sacrificed rats were firstly fixed with 4% polyformaldehyde (PFA). Then they were produced into 3.5 μm sections, respectively. Tissue slides were incubated at 4 °C overnight with anti-VTN mAb (1:50) (Abcam, Cambridge, MA, US), anti-collagen I mAb (1:50) (Abcam, Cambridge, MA, US). Lastly, these sections were observed under a microscope and five random fields were chosen to evaluate the fibrosis score. Scores were measured by the cell cytoplasm staining patterns of the lung tissues as described: score 0, absent staining; score 1, light yellow staining; score 2, light brown staining; score 3, dark brown staining. Fibrosis scores were analyzed by two senior and experienced pathologists in single-blind review and evaluated the pathological changes according to the method proposed by Phillips et al. [21].

### Statistical analysis

All experiments were repeated at least three times in this study. The statistical analysis was performed by using GraphPad Prism 5 software (GraphPad Software, Inc., La Jolla, CA, USA). All data were shown as mean ± standard deviation (SD) unless otherwise noted. The main statistical methods were *t*-*test* and one-way ANOVA. p < 0.05 was considered as statistically significant.

## Results

### Expression level of VTN increased in fibroblasts after received irradiation

**Fig. 1:**
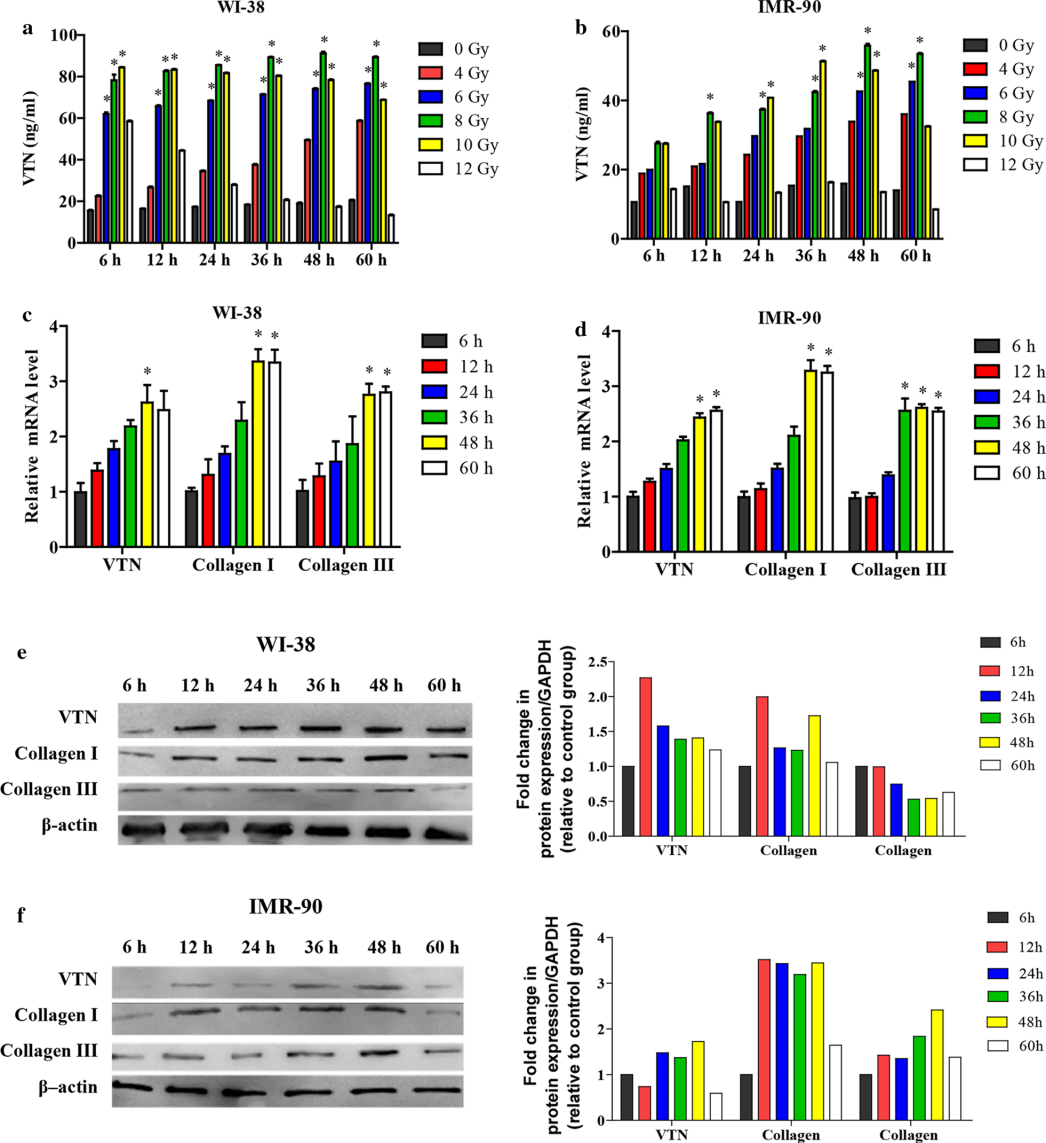
Expression level of VTN increased in irradiated WI-38 and IMR-90 cell lines. **a**, **b** The protein expression levels of VTN at different time after irradiated with different doses in WI-38 (**a**) and IMR-90 cell lines (**b**) were analyzed by ELISA, n = 3, *p < 0.05 vs 0 Gy group. **c**, **d** The mRNA expression levels of VTN, collagen I and III at different time points after 8 Gy irradiation in WI-38 (**c**) and IMR-90 cell lines (**d**) were analyzed by quantitative RT-PCR, n = 3, *p < 0.05 vs 0 h in post-irradiation group. **e**, **f** The protein expression levels of VTN, collagen I and III at different time points after 8 Gy irradiation in WI-38 (**e**) and IMR-90 cell lines (**f**) were analyzed by western blot analysis, β-actin was used to confirm the equal amount of proteins loaded in each lane. The experiments described above were repeated three times at least, and their final data was expressed as mean ± SD

In order to investigate the change in the expression of VTN under irradiation, ELISA assay was undertaken at 6, 12, 24, 36, 48, 60 h after exposure to 0, 4, 6, 8, 10, or 12 Gy of irradiation in WI-38 and IMR-90 cells to detect the secretion level of VTN (Fig. 1a, b). The results exhibited that, it needed 12 h of irradiation with at least dose of 6 Gy for WI-38 cells (p < 0.05) to present increased VTN expression, and IMR-90 cells (p < 0.05) needed at least 8 Gy dose in the same duration for increased VTN expression. In fact, the expression levels of VTN protein reached the highest value after irradiation with 8 Gy for 48 h in both cell lines.

Furthermore, the mRNA and protein expression levels of VTN, collagen I, and collagen III in post-irradiation WI-38 and IMR-90 cells after being treated with 8 Gy dose of irradiation were detected by using quantitative RT-PCR and western blotting, respectively (Fig. 1c–f). The results showed that mRNA expression levels of collagen I and collagen III, which were consistent with the VTN mRNA level, increased with irradiation exposure time and reached the peak at 48 h post-irradiation both in WI-38 and IMR-90 cells (Fig. 1c, d). A similar tendency was revealed in the results of western blot analysis: protein expression levels of VTN, collagen I and collagen III significantly increased with prolonged irradiation time and all the maximal protein expression levels appeared at 48 h post-irradiation (Fig. 1e, f). Pearson analysis (data not shown) showed a positive correlation between protein expression level of VTN and collagen I and III in both WI-38 (r = 0.79, 0.70, respectively; p < 0.01) and IMR-90 cells (r = 0.45, 0.40, respectively; p < 0.05). Considering the changes in protein expression of VTN, collagen I and collagen III induced by irradiation were more obvious in WI-38 cells than that in IMR-90 cells, WI-38 cells were chosen to be treated with irradiation dose of 8 Gy for 48 h as the optimum cell-irradiation condition for our subsequent experiments.

### VTN activated fibrosis regulatory pathways in WI-38 cells

**Fig. 2:**
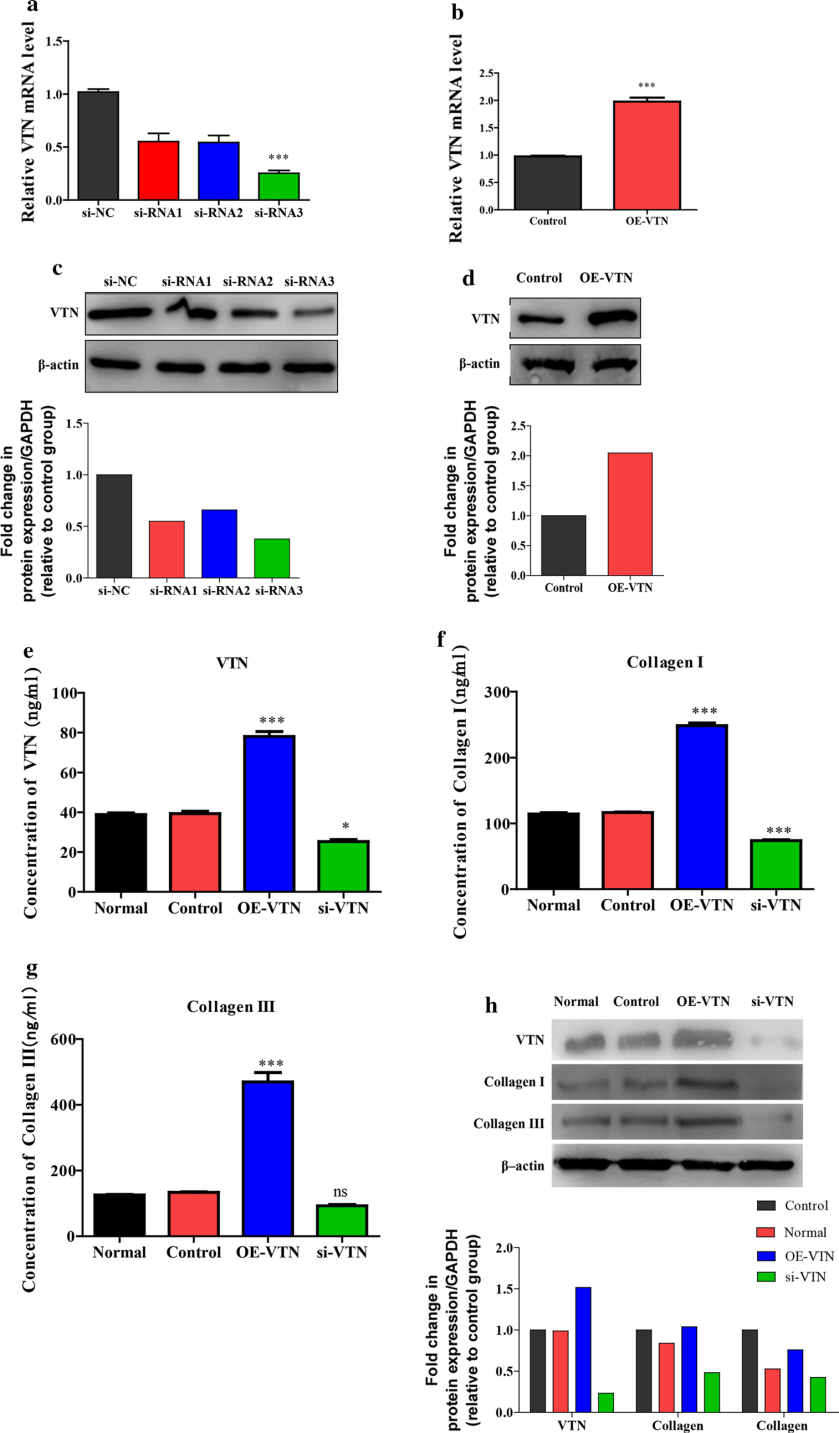
VTN regulated the expression levels of collagen I and III. **a** The transfection efficiency of si-RNA 1 (targeted VTN), si-RNA 2 (targeted VTN) and si-RNA 3 (targeted VTN) were analyzed by quantitative RT-PCR, n = 3, ***p < 0.001 vs the si-NC (the control siRNA to the siRNA targeted VTN) group. **b** The relative mRNA expression levels of VTN in control group and OE-VTN group were analyzed by quantitative RT-PCR, n = 3, ***p < 0.001 vs control. **c** The protein expression level of VTN in the si-NC group, si-RNA groups was analyzed by western blot analysis. **d** The protein expression level of VTN in control group and OE-VTN group were analyzed by western blot analysis. **e** The concentrations of VTN in each group were analyzed by ELISA, n = 3, *p < 0.05, ***p < 0.001 vs the normal group. **f** The concentrations of collagen I in each group were analyzed by ELISA, n = 3, ***p < 0.001 vs the normal group. **g** The concentrations of collagen III in each group were analyzed by ELISA, n = 3, ***p < 0.001 vs the normal group. **h** The protein expression levels of VTN, collagen I and III in different groups were analyzed by western blot analysis. β-actin was used to confirm the equal amount of proteins loaded in each lane. The experiments described above were repeated three times at least, and their final data was expressed as mean ± SD

VTN-overexpressed lentivirus and VTN-si-RNA lentivirus were established and transfected into WI-38 cells to construct VTN-overexpressed (OE-VTN) cells and VTN knockdown cells (si-RNA group). WI-38 cells transfected with non-targeting siRNA (si-NC group) were used as control for si-RNA group, while the mock-vehicle transfected WI-38 cells were applied as the negative control groups for OE-VTN group. VTN overexpressed and si-RNA lentivirus vectors were successfully constructed and verified through qRT-PCR and western blot (Fig. 2a–d). Among the 3 lentivirus vectors designed to interfere with VTN expression, the si-RNA3 had the most effective inhibitory effect on VTN expression in mRNA level compared with si-NC, and was chosen for subsequent construction of VTN silencing cell models. ELISA and western blot analysis were adopted to explore the protein expression levels of VTN, collagen I and III in WI-38 cells in each group at 48 h post-irradiation with 8 Gy. As shown in Fig. 2e–h, in OE-VTN cells, the protein expression levels of VTN were significantly higher than that in the normal and negative control group (p < 0.001). On the contrary, in si-RNA cells, the expression levels of VTN were significantly declined compared with the negative control group. Similarly, the protein expression levels of collagen I and III were significantly up-regulated in OE-VTN cells (p < 0.001 vs negative control), and protein expression level of collagen I was significantly down-regulated (p < 0.01 vs negative control) in si-RNA cells, whereas collagen III did not.

**Fig. 3:**
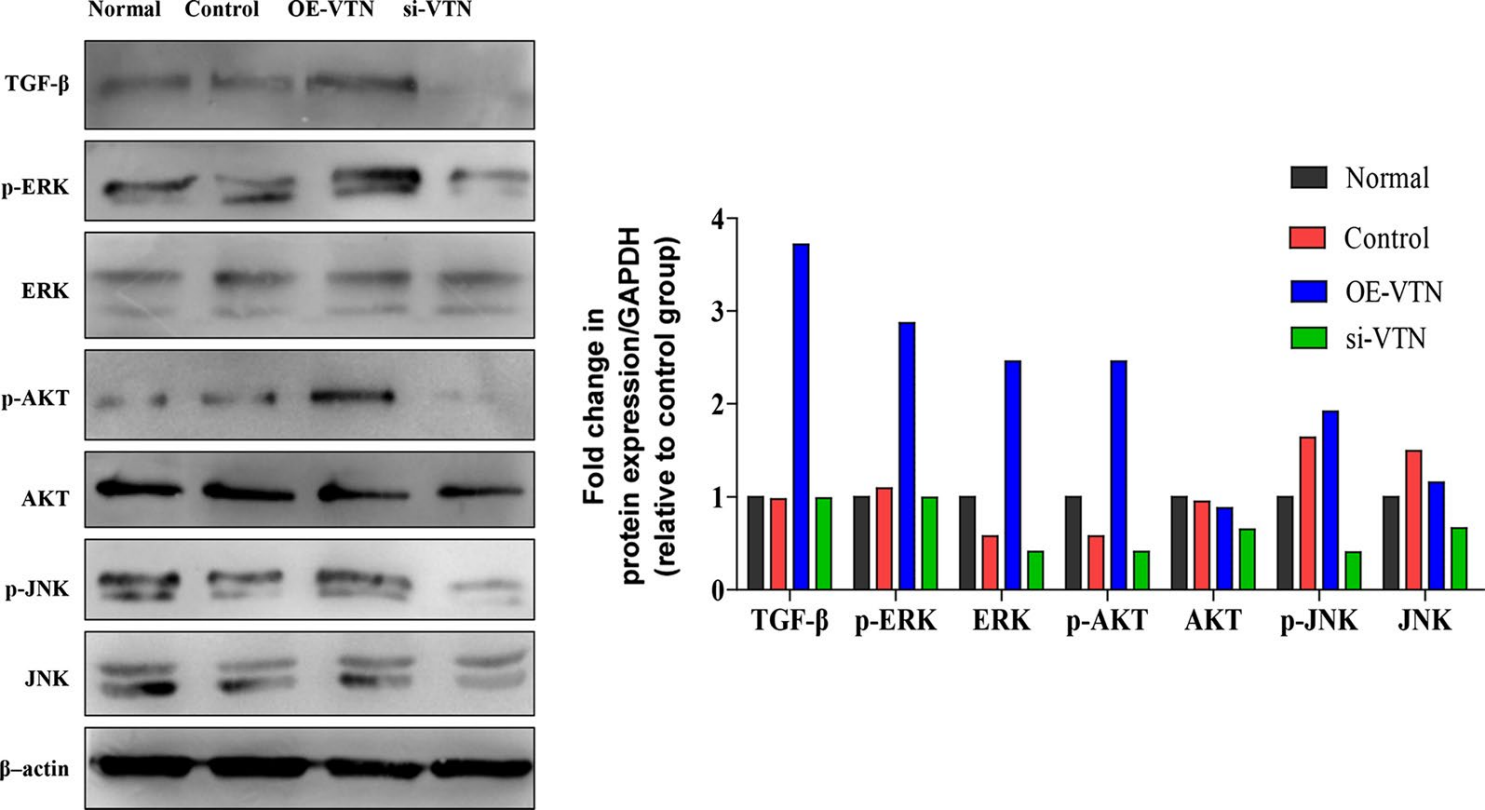
VTN regulated the activation of fibrosis regulatory signal. The protein expression levels of the fibrosis regulatory proteins in the WI-38 cells from different groups were analyzed by western blot analysis, β-actin was used to confirm the equal amount of proteins loaded in each lane. The experiments described above were repeated three times at least, and their final data was expressed as mean ± SD

Many articles have reported that PI3K/AKT, ERK and JNK signaling pathway were involved in the TGF-β1-induced lung fibrosis, and the phosphorylation form of them could act as the key fibrosis regulators [22–26]. Therefore, for further researches, we tested phosphorylation levels of fibrosis regulators, including AKT1, ERK1 and JNK, and utilized western blot analysis to detect the levels of phosphorylated and non-phosphorylated proteins, respectively. Results revealed that the levels of p-AKT and p-ERK were increased (p < 0.05) while the expression level of p-JNK remained almost unchanged and there is no significant change in levels of non-phosphorylated ERK, AKT and JNK compared with control. Conversely, knockdown of VTN decreased the levels of p-AKT, p-ERK, and p-JNK (Fig. 3b). These results indicated that VTN could activate the fibrosis process through activating ERK, AKT and JNK signal pathway in vitro.

### VTN regulated the expression levels of fibrosis-related genes in vivo

**Fig. 4:**
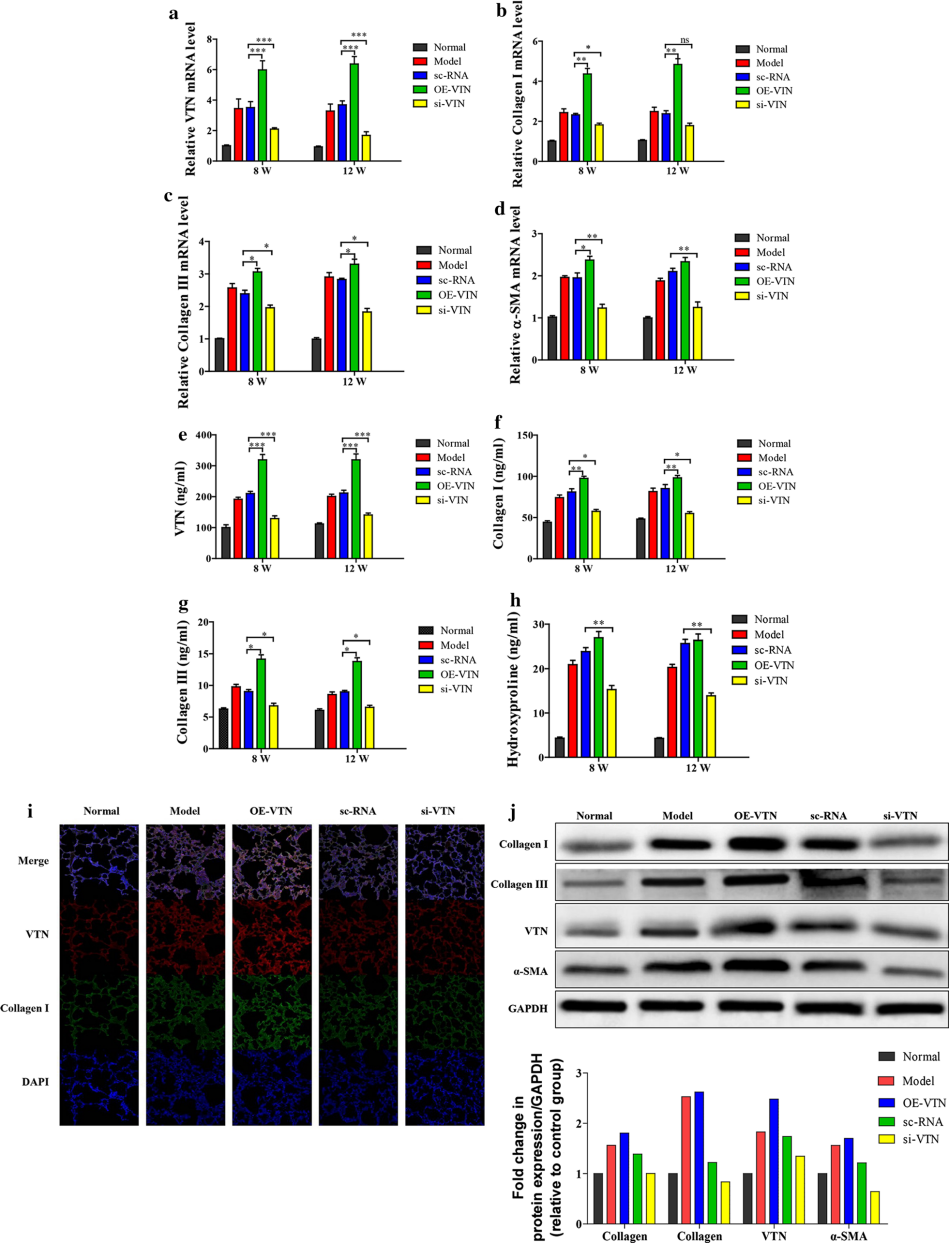
VTN modulated the expression level of fibrosis members in vivo. **a**–**c** The relative mRNA expression levels of VTN (**a**), collagen I (**b**), collagen III (**c**) and α-SMA (**d**) in the lung tissues of mice from different groups were analyzed via qRT-PCR, n = 6, *p < 0.05, **p < 0.01, ***p < 0.001 vs the sc-RNA group (n = 6 mice/group). **e**–**h** The protein concentrations of VTN (**e**), Collagen I (**f**), Collagen III (**g**), and Hydroxyproline (**h**) in the lung tissues of mice from different groups were analyzed by ELISA, n = 6, *p < 0.05, **p < 0.01, ***p < 0.001 vs the sc-RNA group (n = 6 mice/group). **i** The immunohistochemical staining for VTN and collagen I in the lung tissue of mice from different groups; the staining results were imaged in presence of red (VTN), green (collagen I) and blue (nuclei). **j** The protein expression levels of collagen I, collagen III, VTN, and α-SMA in lung tissue of mice from different groups were analyzed by western blot analysis, GAPDH was used to confirm the equal amount of proteins loaded in each lane. The experiments described above were repeated three times at least, and their final data was expressed as mean ± SD

To investigate effects of different expression levels of VTN on RILT process in vivo, VTN overexpression (OE-VTN group, n = 12) and VTN knockdown (si-VTN group, n = 12) mice models were constructed via tail vein injection of VTN overexpressed and interfering lentivirus, the mice injected with empty lentivirus and the same dosage of saline were used as negative control (sc-RNA group, n = 12) and normal group (n = 12), respectively. 48 h after transfection, all the mice in 4 groups were exposed to a single irradiation in a dose of 8 Gy to induce RILT. Meanwhile, mice without irradiation treatment were used as normal group (n = 12). The expression levels of VTN, collagen I, and collagen III in the lung tissues of all mice were demonstrated in Fig. 4. 8 and 12 weeks after irradiation, 6 mice in each group were sacrificed, and their lung tissues were collected for further detection. The mRNA and protein levels of VTN, collagen I and III were increased in the lung tissue from mice in irradiation groups compared with that in normal group (p < 0.05). Compared with the sc-RNA group, the mRNA and protein levels of VTN, collagen I and III were significantly up-regulated in the OE-VTN group (VTN: p < 0.001; collagen I: p < 0.01; collagen III: p < 0.05), which were significantly decreased in si-RNA group (VTN: p < 0.001; collagen I: p < 0.05; collagen III: p < 0.05), except for the mRNA level of collagen I (p > 0.05). Besides, there was no significant difference in the expression patterns of target factors between 8 and 12 weeks post-irradiation. Consistently, immunohistochemical staining results also confirmed the changes in the level of Collagen I in VTN-overexpressed mice (Fig. 4i). Western blot analysis results further verified that VTN induced up-regulation of collagen I, collagen III, and α-SMA (Fig. 4j).

Hydroxyproline is one of the specific amino acids in the collagen and α-SMA is considered as the biomarker in the activation of fibroblasts. Herein, we found that VTN could also affect the expression levels of hydroxyproline and α-SMA (Fig. 4d, h).

### VTN exacerbated fibrotic process in mouse lung

**Fig. 5:**
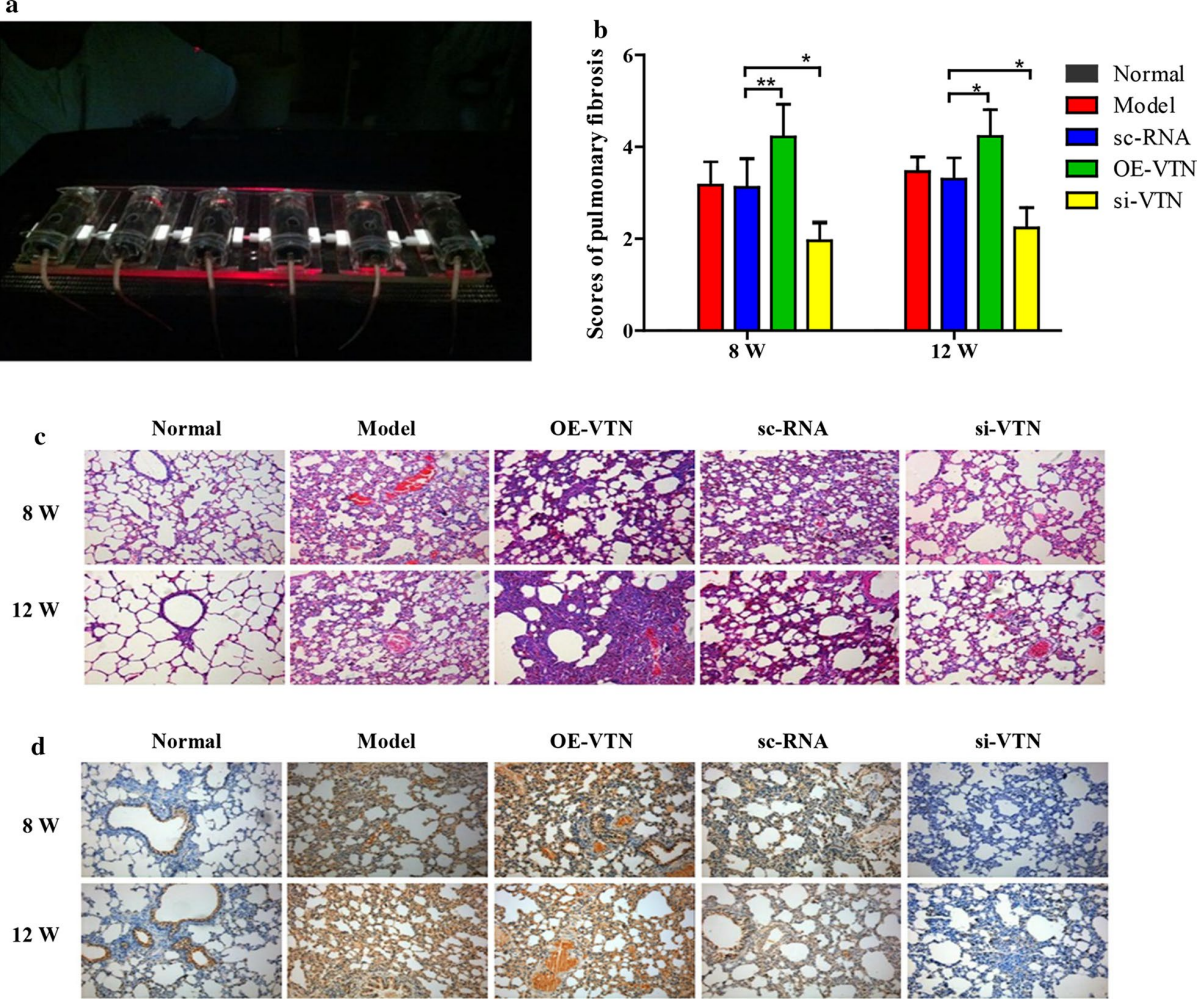
VTN aggravated pulmonary fibrosis. The mice (n = 6 mice/group) in each group were treated with irradiation of 12 Gy for 8 or 12 weeks. Then, the mice in the four irradiated groups and the normal group were sacrificed for the following researches. **a** Irradiation apparatus for the mice. **b** Lung fibrosis scores (the normal group scored 0) are shown as arbitrary units. **c** Effect of VTN on lung inflammation and injury was analyzed by hematoxylin and eosin (H&E) staining, image magnification: ×200. **d** The expression pattern of VTN in irradiation-stimulated mice was analyzed by immunohistochemistry, image magnification: ×200. The experiments described above were repeated three times at least, and their final data was expressed as mean ± SD

With H&E staining, it could be observed that after irradiation for 8 and 12 weeks, some symptoms of RILT that alveolar walls were thickened, alveolar spaces became narrow and parts of the alveolar walls were damaged occurred. In addition, the development and severity of these pathological changes in morphology were aggravated by VTN overexpression in vivo. By contrast, the process was slower and less severe in the si-VTN group compared with model group (Fig. 5c). The immunohistochemistry of VTN was demonstrated in Fig. 5d. Through microscopical observation, the degrees of pulmonary fibrosis in 5 groups were demonstrated in Fig. 5b (the normal group scored 0). Significantly higher fibrosis extent in OE-VTN group (p < 0.05) and obviously lower fibrosis level in si-RNA group (p < 0.05) were found, when compared with sc-RNA. These results suggested that VTN could exacerbate fibrotic process in mice lung after irradiation.

## Discussion

VTN is a type of secreted proteins, which exists in serum and tissues with two kinds of types, namely single chain or double chains. Accumulating evidences clearly indicated that VTN is closely involved in blood coagulation, fibrinolysis, complement activation, cell adhesion and migration [10–12]. Carpagnano et al. reported the increased expression of VTN in the exhaling gas condensate of patients with interstitial lung disease [13]. Moreover, Courey et al. reported that the combination of VTN and PAI-1 could aggravate pulmonary fibrosis. All the above results suggested the involvement of VTN in pulmonary fibrosis [16]. Other researchers have also demonstrated higher expression level of VTN in fibrosis tissues [14, 17, 27], this indicates that VTN might involve in the fibrotic process, while the underlying mechanisms remain unknown. Moreover, the research of the relationship between VTN and RILT is still rarely reported. Our previous works showed that elevated basal expression of VTN was related to RILT [9], indicating that VTN might play an important role in the process of RILT.

Pulmonary fibrosis is a late stage of the RILT, and is diagnosed with excessive fibroblast proliferation, collagen and extracellular matrix (ECM) deposition. Fibroblast is the key effector cells in pulmonary fibrosis, once the process was activated, they would transform into myofibroblasts to express α-SMA as typical characteristic and secrete ECM components such as collagens and fibronectins [8], which are the major contributors of pulmonary fibrosis. The in vitro experiment in current study revealed increased protein and mRNA expression levels of VTN in the fibroblasts after irradiation, and there was a positive correlation between the expression level of VTN and collagen I and collagen III. Masaharu et al. revealed that VTN had potential protection roles in human umbilical vein endothelial cell after radiation injury, but they have not detected the high expression of VTN in cells before and after radiation [28]. The VTN receptor, integrins a_ν_b_5_ and a_ν_b_3_, were also found to be up-regulated in malignant glioma cells after photon radiation [29]. The present study firstly demonstrated the high expression of VTN in the fibroblasts after irradiation. Previous researches have showed that there existed interaction between VTN and collagen, which could be affected by aggregation of collagen and the multimerization of VTN [30]. Herein, we found that the expression of VTN was positively correlated with collagens’ expression and VTN had regulation effects on collagens.

Our further study revealed that expression of VTN could not only mediate the regulation of collagens, but also increase expression levels of TGF-β, and phosphorylation levels of ERK, AKT, and JNK. TGF-β, a kind of chemotactic factor secreted by fibroblast, myofibroblast, and macrophage, is a potent paracrine mediator of myofibroblast differentiation and contributes to the development of pulmonary fibrosis after the expansion of lung myofibroblasts [31–33]. To be exact, TGF-β was considered as a vital cause for radiation-induced fibrosis, as its high expression in the impaired skin and muscular fibrotic tissues [34]. Suppressing the expression of TGF-β was also reported to alleviate radiation induced muscular fibrosis [35]. Of note, it has been proved that VTN could interact with various growth factors, including TGF-β [31, 36, 37]. Additionally, up-regulation of α-SMA expression and increase of collagen gel contraction induced by TGF-β are VTN dependent [38, 39]. However, this study demonstrated that expression of VTN can regulate the TGF-β to some extent.

PI3K, AKT1, ERK1 and JNK had been reported to be the downstream signals of TGF-β. An ex vivo study found that inhibiting PI3K/Akt was able to block cell proliferation, increase of α-SMA expression and collagen production induced by TGF-β, which demonstrated the dominant roles of the PI3K/Akt pathway in the proliferation and differentiation process of lung fibroblast [40]. In addition, Caraci et al. demonstrated that TGF-β1 induced α-SMA expression and collagen production in human lung fibroblasts via ERK1/2 pathway activation, GSK-3β inhibition, and nuclear β-catenin translocation [41]. Utsugi et al. [42] suggested that TGF-β1-induced connective tissue growth factor (CTGF) mRNA expression was mediated through the JNK-dependent pathway [39]. Our current study showed that increased VTN promoted the phosphorylated levels of ERK, AKT and JNK, which indicated that VTN overexpression activated the fibrosis regulatory pathway dominated by TGF-β to further promote the production of collagens. In the in vivo study, the mice infected with VTN-overexpressed lentivirus exhibited enhanced TGF-β1 production which coupled with increased expression of α-SMA. In contrast, the mice infected with VTN-si-NRA lentivirus decreased TGF-β1 and suppressed transcription of α-SMA. These results, together with the data mentioned above, suggested that VTN might promote the differentiation of lung fibroblasts to form a myofibroblast phenotype through up-regulation expression of TGF-β1 and increasing the transcription of α-SMA.

## Conclusions

In conclusion, our study showed that VTN played an important role in RILT via up-regulating the expression of collagen, activating the fibrosis regulatory pathway to promote lung fibrosis. These findings identified a novel mechanism that contributes to lung fibrosis and suggested that VTN can be a promising therapeutic target for RILT.

## Additional file

**Additional file 1: Table S1.** Primer sequences for quantitative RT-PCR. **Table S2.** Primer sequences for construction of VTN-overexpressed and VTN-interfering vectors.

## Abbreviations

RILT: radiation-induced lung toxicity
VTN: vitronectin
RILT: radiation-induced lung toxicity
ATCC: American type culture collection
mAb: monoclonal antibody
qRT-PCR: quantitative RT-PCR
H&E: hematoxylin and eosin
PFA: polyformaldehyde
SD: standard deviation
ECM: extracellular matrix
CTGF: connective tissue growth factor
