# Supplementary material for: The positive role of vitronectin in radiation induced lung toxicity: the in vitro and in vivo mechanism study
Source: J Transl Med. 2018 Apr 16;16:100. doi: 10.1186/s12967-018-1474-y (PMC5902986; doi:10.1186/s12967-018-1474-y)
Supplement: Supplementary file 1 — Additional file 1: Table S1. Primer sequences for quantitative RT-PCR. Table S2. Primer sequences for construction of VTN-overexpressed and VTN-interfering vectors. [file 12967_2018_1474_MOESM1_ESM.doc]

**Effects of vitronectin on** **radiation induced lung toxicity: *in vitro* and *in vivo* study**

**Additional file 1: Table S1. Primer sequences for quantitative RT-PCR**

**Abbreviations: VTN=** vitronectin.

| **gene** | **primers** | **products（bp）** |
| --- | --- | --- |
| **VTN** | 5- TACCCCAAGCTCATCCGAGA -3  5- AGGACACCATCCTCAAAGCG -3 | 131 |
| **ERK** | 5- TGACCTCAAGCCTTCCAACC -3  5- GCCAGAATGCAGCCTACAGA -3 | 213 |
| **TGF-β** | 5- GGGCTACCATGCCAACTTCT -3  5- GCACGATCATGTTGGACAGC -3 | 209 |
| **PTK2** | 5- CAACCACCTGGGCCAGTATT -3  5- AGCAGGCCACATGCTTTACT -3 | 102 |
| **JUN** | 5- GCTGCGTTAGCATGAGTTGG -3 | 176 |
| **AKT1** | 5- ACTGTCATCGAACGCACCTT -3  5- CTCCTCCTCCTCCTGCTTCT -3 | 108 |
| **P13K** | 5- TGATAGGGGGAATTGGAGGC -3  5- TGTTGACGGGTGTAGGTCC -3 | 114 |
| **GAPDH** | 5- CTGGGCTACACTGAGCACC -3  5- AAGTGGTCGTTGAGGGCAATG -3 | 101 |

**Additional file 1: Table S2. Primer sequences for construction of VTN-overexpressed and VTN-interfering vectors**

**Abbreviations: si-NC=Non-specific control siRNA; VTN-OE= vitronectin overexpression.**

| **Name** | **primers** |
| --- | --- |
| **si-NC** | 5- UUCUCCGAACGUGUCACGUTT -3  5- TTAAGAGGCUUGCACAGUGCA -3 |
| **VTN-siRNA1** | 5- CCAGAUUCAUCAUCAAUGATT -3  5- TTGGUCUAAGUAGUAGUUACU -3 |
| **VTN-siRNA2** | 5- GGCACACCAAAGGUAUGAUTT -3  5- TTCCGUGUGGUUUCCAUACUA -3 |
| **VTN-siRNA3** | 5- GGCACACCAAAGGUAUGAUTT -3  5- TTCCGUGUGGUUUCCAUACUA -3 |
| **VTN-OE** | 5- CCGGAATTCATGGCACCCCTGAGACCCCTTCT -3  5- CGCGGATCCCTACAGATGGCCAGGAGCTGG -3 |
